# Interconnected pathways and emerging therapies in chronic kidney disease and heart failure: A comprehensive review

**DOI:** 10.1002/ehf2.15345

**Published:** 2025-06-18

**Authors:** Johann Bauersachs, Eri Toda Kato, Janani Rangaswami

**Affiliations:** ^1^ Department of Cardiology and Angiology Hannover Medical School Hannover Germany; ^2^ Department of Cardiovascular Medicine and Institute for Advancement of Clinical and Translational Science Kyoto University Hospital Kyoto Japan; ^3^ George Washington University Washington DC USA

**Keywords:** Chronic kidney disease, Chronic heart failure, Glucagon‐like peptide‐1 receptor agonist, Mineralocorticoid receptor antagonist, Pathogenesis, Sodium‐glucose cotransporter 2 inhibitor

## Abstract

Chronic kidney disease (CKD) and chronic heart failure (HF) frequently coexist and, when comorbid, are associated with poorer outcomes. These two diseases have common risk factors, such as diabetes, obesity and hypertension, and common pathophysiological connected mechanisms, including inflammation, endothelial dysfunction, neurohormonal activation and fibrosis. Early diagnosis and intervention are important to slow CKD progression and reduce HF events. Shared therapeutic targets for CKD and HF include the renin–angiotensin system (RAS), sodium‐glucose cotransporter 2 (SGLT2), mineralocorticoid receptor (MR) and glucagon‐like peptide‐1 (GLP‐1) receptor. For the management of CKD, current treatment guidelines recommend the use of RAS inhibitors, SGLT2 inhibitors, the nonsteroidal MR antagonist finerenone and GLP‐1 receptor agonists. Challenges in the management of patients with CKD and HF include the presence of other comorbidities, leading to polypharmacy. This review highlights gaps and opportunities for improving the management of patients with CKD and chronic HF.

## Graphical abstract

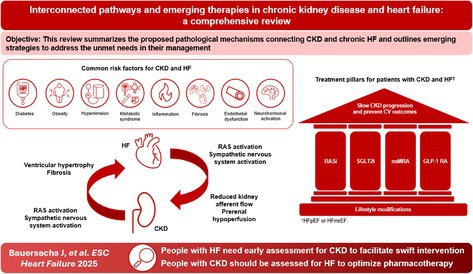

## Introduction

Chronic kidney disease (CKD) and heart failure (HF) frequently coexist,^1^, ^2^, ^3^ exacerbating both conditions,^4^ and contributing to significant mortality.^5^ The prevalence and burden of these conditions are rising. In 2017, CKD affected an estimated 9.1% of the global population, impacting 697.5 million people worldwide and contributing to 1.2 million deaths.^6^ HF affects more than 64 million people worldwide with a prevalence of 1%–3% in the general adult population.^7^

In HF, distinct epidemiological patterns are observed across the ejection fraction (EF) spectrum. Data from the European Society of Cardiology (ESC) HF Long‐Term registry reveal that HF with reduced EF (HFrEF; EF ≤40%) accounted for 62% of cases, while HF with preserved EF (HFpEF; EF ≥50%) represented 24% of the HF population.^8^ The HFrEF population was predominantly male (76%), with a median age of approximately 66 years, while the HFpEF population was 50% female, with a median age of 72 years (interquartile range, 62–80).^8^ The intermediate phenotype, HF with mildly reduced EF (HFmrEF; EF 41%–49%), accounted for 14% of cases and demonstrated demographic characteristics between the other two categories.^8^

Due to shared risk factors and pathogenic mechanisms, CKD and HF are interlinked, with almost one‐quarter of patients aged >65 years with CKD having HF^1^ and approximately 40%–50% of patients with HF having CKD.^2^, ^3^ Comorbid CKD is associated with poorer outcomes for patients with HF.^3^, ^9^

Despite recent advances in therapeutic management, the burden of CKD and HF remains substantial.^7^, ^10^ One‐year and 5‐year mortality rates in patients with HF are ~15%–30% and ~50%–75%, respectively.^7^ In patients with HF with CKD, there is a greater risk of death than in those with HF without CKD: 49% greater in those with HFrEF, 51% greater in those with HFmrEF, and 32% greater in those with HFpEF.^11^ CKD is also expected to become the fifth‐leading cause of death by 2040.^10^

The objectives of this article are to review the proposed pathological mechanisms that connect CKD and chronic HF, to identify the unmet needs for CKD and chronic HF treatment management, and to outline emerging strategies to address these unmet needs.

## Evaluation of kidney function in patients with HF

**Figure 1:**
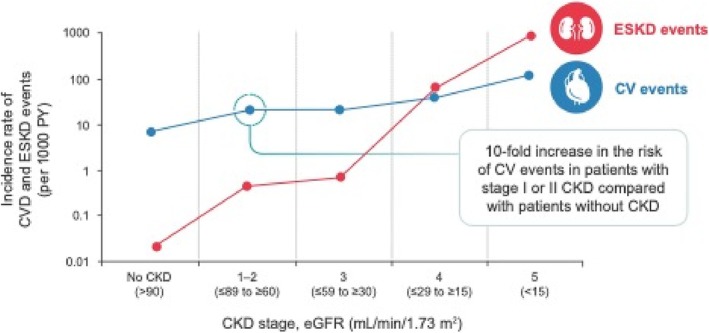
Incidence of ESKD and CV events according to baseline CKD stage. Figure uses a logarithmic scale. CKD, chronic kidney disease; CV, cardiovascular; CVD, cardiovascular disease; eGFR, estimated glomerular filtration rate; ESKD, end‐stage kidney disease; PY, patient‐years. Adapted from de Jong P, *et al.* *Nephron Clin Pract* 2009;111:c204–c211.^13^

Early diagnosis of CKD is important to improve HF outcomes, as the risk of cardiovascular (CV) events increases as estimated glomerular filtration rate (eGFR) declines.^12^ The risk of CV events is approximately 10‐fold higher in patients with stage I or II CKD compared with those without CKD (*Figure* *1*).^13^

The Kidney Disease: Improving Global Outcomes (KDIGO) guidelines define CKD based on the following criteria being present for more than 3 months: eGFR <60 mL/min/1.73 m^2^ or markers of kidney damage (one or more structural or functional abnormalities, albuminuria [urine albumin‐to‐creatinine ratio ≥30 mg/g {≥3 mg/mmol}], urine sediment abnormalities, persistent haematuria, electrolyte and other abnormalities due to tubular disorders, abnormalities detected by histology, structural abnormalities detected by imaging or history of kidney transplantation).^12^

CKD is often underdiagnosed in patients with HF.^14^, ^15^ A study using data from the electronic health records of the US Department of Veterans Affairs healthcare system (*N* = 1 446 053) showed that individuals with HF and CKD, defined according to the KDIGO criteria, had a high risk of death and hospitalization, most of which was not explained by abnormal kidney structure or function.^16^ These findings highlight the need to examine whether the definition of CKD using a single eGFR measurement or a combination of eGFR and urine albumin‐to‐creatinine ratio (UACR) measurements is optimal for those with co‐existing diseases.

Early kidney disease is typically asymptomatic, therefore laboratory testing is usually required for diagnosis,^17^ and guidelines recommend annual screening of both eGFR and UACR for detecting CKD in patients with HF.^12^, ^18^ Albuminuria, a powerful marker for early kidney damage, is measured using UACR. Of note, CKD may be present even when eGFR levels are normal^12^; an abnormal UACR value alone is diagnostic of CKD if present for longer than 3 months.^12^ In addition to being a marker for kidney damage, albuminuria can be a powerful marker for CV disease (CVD). Elevated UACR is a risk factor for increased CV events and incident and progression of HF, as well as an early independent risk factor for incident CKD.^19^, ^20^ In addition, each 50% reduction in UACR in the first 6 months can reduce HF risk by 27%^21^ and a 30% reduction in 6‐month UACR can reduce risk of CKD progression by 32%.^22^

The addition of UACR screening to traditional CVD risk factor assessments improves the prediction of incident HF in people without a history of CVD,^23^, ^24^ illustrating the value of early UACR screening. Assessing an individual's UACR level requires a morning midstream urine sample^12^; this is easy for both the patient and physician and can be performed in routine clinical practice. However, US and international cohort studies have demonstrated that UACR testing rates are low (4%–53%) in people with diabetes and/or hypertension (the most common risk factors for both CKD and HF), in contrast to eGFR testing rates, which are consistently high (approximately 90%).^25^, ^26^, ^27^, ^28^ The lack of UACR testing potentially leaves many patients with HF with undiagnosed and untreated CKD.

## Diagnosis of HF in patients with CKD

Clinical HF is suspected based on clinical history, typical signs and symptoms, risk factors and abnormalities on a cardiac echogram or electrocardiogram.^29^, ^30^ Measurement of natriuretic peptides is useful to support a diagnosis or exclusion of HF; in stable patients, a concentration of brain natriuretic peptides ≥35–40 pg/mL or N‐terminal pro brain natriuretic peptides (NT‐proBNP) ≥125 pg/mL is suggestive of HF.^29^, ^30^, ^31^ Measurement of brain natriuretic peptides or NT‐proBNP is also recommended at admission in patients hospitalized for HF to establish a prognosis.^29^ Transthoracic echocardiography is recommended during initial evaluation to assess cardiac structure and function, and to evaluate left ventricular EF, defining the HF phenotype.^29^, ^30^

The NT‐proBNP concentration predicts a greater absolute risk of adverse outcomes in patients with HF and reduced kidney function versus those with preserved kidney function.^32^ One study demonstrated that as eGFR declined, absolute levels of NT‐proBNP increased in patients with CKD and HF; this trend was greater in patients with CKD at baseline.^32^ NT‐proBNP increased by 9%, 8% and 23% per 10 mL/min/1.73 m^2^ decline in eGFR in people with baseline eGFR ≥60, 45–60, and <45 mL/min/1.73 m^2^, respectively (*P* for nonlinearity <0.001). Each doubling in NT‐proBNP was associated with a 37% relative increase in the primary endpoint of hospitalization for HF or CV death (hazard ratio [HR], 1.37; 95% confidence interval [CI], 1.34–1.41), which was consistent across baseline eGFR categories (*P*‐interaction = 0.42). For the same incidence of the primary outcome, NT‐proBNP levels were approximately 2.5‐ to 3.5‐fold lower in people with eGFR <45 mL/min/1.73 m^2^ compared with those with eGFR ≥60 mL/min/1.73 m^2^.

## Pathogenesis of CKD and HF

**Figure 2:**
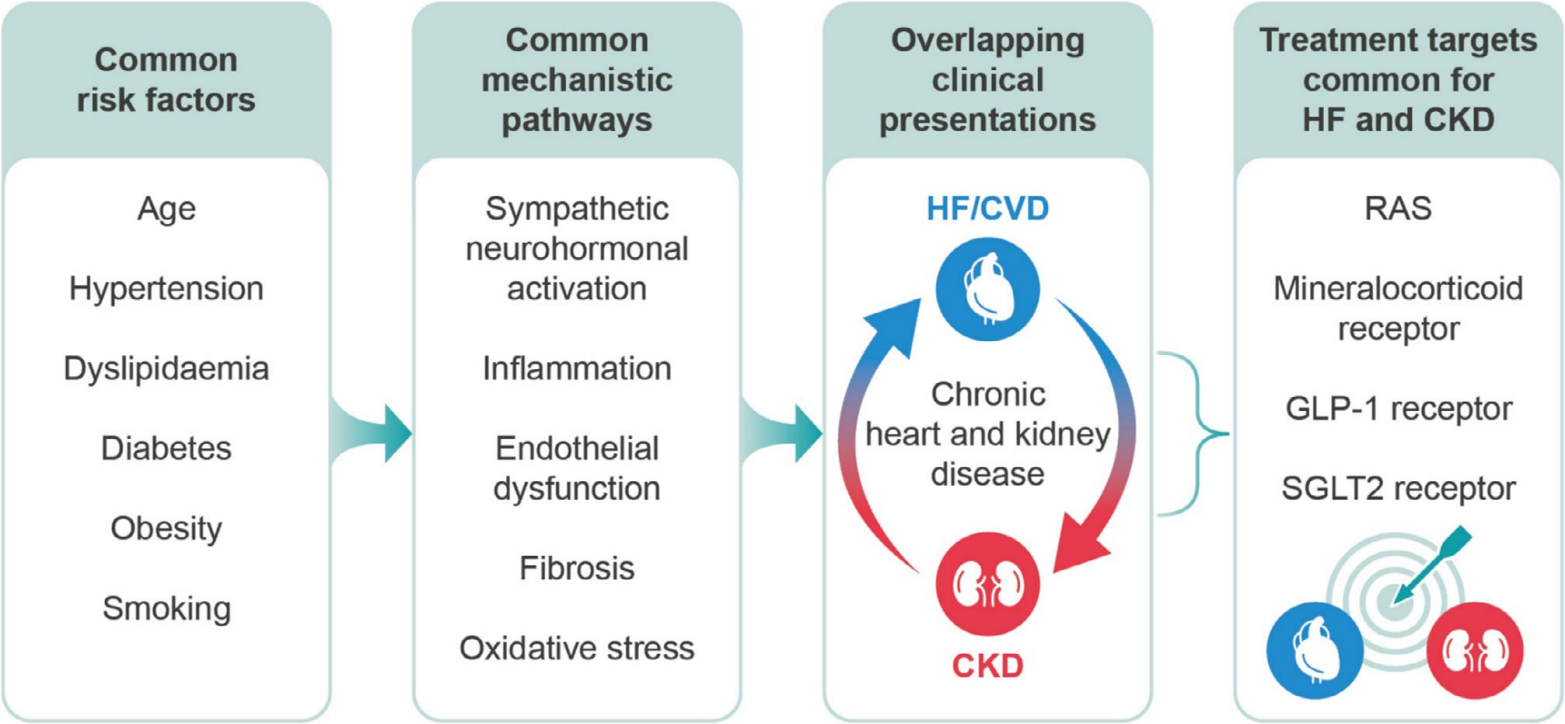
Shared risk factors and pathogenic mechanisms of CKD and HF.^33^, ^34^ CKD, chronic kidney disease, CVD, cardiovascular disease, GLP‐1, glucagon‐like peptide‐1; HF, heart failure, RAS, renin–angiotensin system; SGLT2, sodium‐glucose cotransporter 2.

Within the failing kidneys and heart, common pathophysiological mechanisms such as sympathetic neurohormonal activation, inflammation, endothelial dysfunction, fibrosis and oxidative stress converge over time and promote organ damage and dysfunction (*Figure* *2*).^33^ Shared risk factors that predispose this pathophysiology include diabetes, obesity, metabolic syndrome and hypertension.^33^, ^35^

HF initiates a complex cascade of physiological responses when systolic and/or diastolic dysfunction impairs cardiac output. Initially, the body activates compensatory mechanisms, primarily through the renin–angiotensin system (RAS) and sympathetic nervous system. However, these adaptations often prove insufficient, leading to reduced kidney afferent flow and subsequent prerenal hypoperfusion.^36^ This triggers additional compensatory responses, including further activation of both the sympathetic nervous system and the RAS. While these mechanisms attempt to maintain organ perfusion, their chronic activation becomes maladaptive, contributing to both HF progression and CKD.^33^, ^36^ The sustained activation of these pathways promotes fluid retention and oxidative stress, resulting in adverse cardiac remodelling characterized by ventricular hypertrophy and fibrosis.^4^, ^33^, ^37^ This creates a detrimental feedback loop: HF causes kidney congestion and hypoperfusion, which deteriorates kidney function, while worsening kidney function further exacerbates HF.^4^ This bidirectional relationship establishes a self‐perpetuating cycle between CKD and HF, where each condition accelerates the progression of the other.^4^

Chronic systemic inflammation is the central pathogenic mechanism in both CKD and HF driven by multiple factors.^35^ In patients with HFrEF, cardiomyocyte damage from infection, ischaemia or toxicity can trigger an inflammatory response, while risk factors such as obesity, diabetes and hypertension can trigger inflammation in patients with HFpEF.^35^ CKD exacerbates this process, interacting synergistically with obesity and diabetes to intensify the inflammatory response in the heart,^35^ further highlighting the shared pathogenesis of CKD and HF. Persistent local and systemic inflammatory responses impair microvascular response to vasoactive mediators in the kidney, and subsequently promote arterial calcification, endothelial dysfunction and tubulointerstitial fibrosis through activation of RAS.^35^

Inappropriate mineralocorticoid‐receptor (MR) activation is increasingly recognized as a pivotal mechanism for increased production of proinflammatory cytokines, oxidative stress, endothelial dysfunction and ultimately fibrosis.^38^, ^39^ In people with insulin resistance, obesity, diabetes, dyslipidaemia or metabolic syndrome, several pathways can result in MR overactivation or ‘inappropriate’ MR activation, including changes in MR expression, and modulation of the MR by aldosterone and cortisol.^37^ The downstream effects of MR overactivation include increased myocardial stiffness, impaired left ventricular relaxation, glomerular and interstitial fibrosis in the kidneys, a decline in eGFR, proteinuria and progressive loss of kidney function.^35^, ^38^, ^40^, ^41^ One emerging hypothesis identifies the cause of MR activation as excess levels of aldosterone, caused by adipocytes that secrete aldosterone or aldosterone‐secreting factors that target the adrenal gland and promote the generation of aldosterone. Increased aldosterone levels have been observed in individuals with metabolic syndrome and obesity.^35^ Aldosterone may trigger a cascade of mechanisms that typically leads to fibrosis in the heart, vessels and kidneys.^33^ Activation of RAS and the adrenergic system may also contribute to progressive remodelling and contractile dysfunction in patients with HFpEF, as well as impaired natriuresis, and abnormal salt and water retention resulting in extracellular volume expansion and the development of hypertension associated with CKD.^34^, ^35^

## Proposed mechanisms of action of therapies for managing CKD and HF

Given the shared pathophysiology of CKD and HF, several treatment targets are common for both diseases (*Figure* *2*), including the RAS, sodium‐glucose cotransporter 2 (SGLT2), MR and glucagon‐like peptide‐1 (GLP‐1) receptor.

RAS inhibitors (RASis; angiotensin‐converting enzyme inhibitors or angiotensin‐receptor blockers) and angiotensin receptor‐neprilysin inhibitors (ARNIs) are beneficial for the management CKD and HF as they can suppress vasoconstriction and reduce blood pressure.^42^, ^43^, ^44^ Similarly, SGLT2 inhibitors (SGLT2is) reduce the risk of CKD and HF through multiple physiological mechanisms; these include amelioration of tissue hypoxia, regulation of body weight, promotion of diuresis through glucosuria and natriuresis, and reduction of systemic blood pressure. These combined mechanisms decrease the pathophysiological burden on both kidney and cardiac tissues.^45^ MR antagonists (MRAs) reduce fibrosis and oxidative stress, improve endothelial function and decrease inflammation in the kidneys and heart.^40^, ^41^ GLP‐1 receptor agonists (GLP‐1 RAs) increase glucose‐induced insulin secretion and were developed to improve glycaemic control in patients with type 2 diabetes (T2D).^46^ The body weight loss induced by these therapies may reduce hyperfiltration associated with T2D and obesity^47^ and may have anti‐inflammatory and antifibrotic effects.^48^, ^49^

## Standard of care for patients with CKD and HF

Current treatment guidelines recommend RASis and SGLT2is to slow CKD progression and to reduce the risk of HF in patients with CKD.^12^, ^30^, ^50^, ^51^, ^52^ Finerenone (a nonsteroidal MRA) is also recommended in patients with T2D and CKD with albuminuria.^12^, ^50^, ^51^, ^52^ In addition, GLP‐1 RAs are recommended in patients with T2D and established CKD or CVD for reducing CV risk and/or lowering glucose levels.^51^, ^53^ A pillared approach to treatment, in addition to lifestyle modifications, has been recommended to slow progression of CKD and prevent CV outcomes.^54^, ^55^ This approach for the management of CKD is similar to the quadruple‐therapy approach currently recommended for the treatment of HFrEF (early initiation of beta blockers, ARNIs, MRAs and SGLT2is).^29^, ^56^ Indeed, an actuarial analysis based on data from 12 large randomized clinical trials, encompassing over 87 000 patients with T2D and at least moderately increased albuminuria, supports the implementation of a four‐pillared approach (RASis, SGLT2is, a nsMRA and GLP‐1 RAs) in this population; this strategy was associated with a 4.4% absolute risk reduction in major adverse CV events (nonfatal myocardial infarction, nonfatal stroke or CV death) over 3 years.^57^ Across several age ranges, this quadruple‐therapy approach was estimated to lead to substantial reductions in CV and kidney outcomes and improvements in event‐free and overall survival for patients with T2D and CKD.^57^ For example, a patient initiating combination therapy at 50 years of age was estimated to gain 3.2 years in major adverse CV event‐free survival compared with standard care.^57^ Current treatment guidelines do not yet provide recommendations or algorithms on whether therapies should be administered simultaneously or sequentially; however, it is important that patients receive timely treatment of all therapeutic pillars.

Despite the substantial burden associated with these co‐existing diseases, very few studies only enrol patients with an eGFR <30 mL/min/1.73 m^2^ and HF.^58^, ^59^ While subgroup analyses are available from CKD (including analyses of participants with HF) and HF trials (including analyses of participants with CKD),^53^, ^60^, ^61^, ^62^, ^63^, ^64^, ^65^, ^66^, ^67^, ^68^, ^69^, ^70^, ^71^, ^72^, ^73^, ^74^, ^75^ they are often exploratory and insufficiently powered with respect to efficacy outcomes. Collectively, this creates gaps in the management of patients with both CKD and HF.

The main findings of studies of RASis/ARNIs, SGLT2is, MRAs and GLP‐1 RAs are summarized in *Tables* *1*, *2*, *3*, *4* and described below.

**Table 1:** Key findings of RASi randomized clinical trials in patients with CKD or HF

Trial	Drug	Trial population (*N*)^a^	Key inclusion criteria	CKD outcomes (vs. placebo)	HF outcomes (vs. placebo)
*Participants with CKD*					
RENAAL^76^	Losartan	CKD (*N* = 1513)	UACR ≥300 mg/L and a serum creatinine value between 1.3 and 3.0 mg/dL (with a lower limit of 1.5 mg/dL for male patients weighing >60 kg)	Primary outcome (composite of a doubling of the baseline serum creatinine concentration, ESKD, or death): RR, 16% (95% CI, 2%–28%); *P* = 0.02	First hospitalization for HF: RR, 32%; *P* = 0.005
IDNT^77^	Irbesartan	Hypertension and nephropathy due to T2D (*N* = 1715)	Urinary protein excretion of ≥900 mg/24 hours and a serum creatinine concentration between 1.0 and 3.0 mg/dL in women, and between 1.2 and 3.0 mg/dL in men	Primary outcome (composite of a doubling of the baseline serum creatinine concentration, ESKD, or death): aRR, 0.81 (95% CI, 0.67–0.99); *P* = 0.03	Hospitalization for HF: aRR, 0.77
Meta‐analysis^78^	ACEis and ARBs	CKD (*N* = 64 768)	CKD	Primary outcome (kidney failure): ACEis: OR, 0.61 (95% credible interval, 0.47–0.79) ARBs: OR, 0.70 (95% credible interval, 0.52–0.89)	ACEis: OR, 0.82 (95% credible interval, 0.71–0.92) ARBs: OR, 0.76 (95% credible interval, 0.62–0.89)
HARP‐III^79^	S/V vs. irbesartan	CKD (*N* = 414)	eGFR ≥45 and <60 mL/min/1.73 m^2^ and UACR >20 mg/mmol or eGFR of ≥20 and <45 mL/min/1.73 m^2^ (regardless of UACR)	Primary outcome (change in eGFR at 12 months): difference in means, −0.1 (SE, 0.7) mL/min/1.73 m^2^ Change in UACR: average difference in means, −9% (95% CI, −18 to −1)	NT‐proBNP: reduction, 18% (95% CI, 11%–25%)
*Participants with HF*					
HEAAL^80^	Losartan	HFrEF (*N* = 3846)	LVEF ≤40%		Primary outcome (composite of death or hospitalization for HF): HR, 0.90 (95% CI, 0.82–0.99); *P* = 0.027^b^ Death: HR, 0.94 (95% CI, 0.84–1.04); *P* = 0.24^b^ Hospitalization for HF: HR, 0.87 (95% CI, 0.76–0.98); *P* = 0.025^b^
I‐PRESERVE^81^	Irbesartan	HFpEF (*N* = 4128)	LVEF ≥45%		Primary outcome *(*composite of all‐cause death or hospitalization for CVD): HR, 0.95 (95% CI, 0.86–1.05); NS Hospitalization for HF: HR, 0.95 (95% CI, 0.81–1.10); NS
PARADIGM‐HF^82^, ^83^	S/V vs. enalapril	HFrEF (*N* = 8399)	LVEF ≤40%	Change in eGFR (mL/min/1.73 m^2^)/year: S/V, 1.61 (95% CI, 1.77–1.44) vs. enalapril, 2.04 (95% CI, 2.21–1.88); *P* < 0.001 Change in UACR (mg/mmol): S/V, 1.20 (95% CI, 1.04–1.36) vs. enalapril, 0.90 (95% CI, 0.77–1.03); *P* < 0.001	Primary outcome (composite of CV death or hospitalization for HF): HR, 0.80 (95% CI, 0.73–0.87); *P* < 0.001 <60 eGFR (mL/min/1.73 m^2^) at baseline: HR, 0.79 (95% CI, 0.69–0.90); ≥60 eGFR (mL/min/1.73 m^2^) at baseline: HR, 0.81 (95% CI, 0.73–0.91), *P* = 0.70 Hospitalization for HF: HR, 0.79 (95% CI, 0.71–0.89); *P* < 0.001 <60 eGFR (mL/min/1.73 m^2^) at baseline: HR, 0.79 (95% CI, 0.67–0.95); ≥60 eGFR (mL/min/1.73 m^2^) at baseline: HR, 0.81 (95% CI, 0.70–0.94); *P* = 0.83
PARAGON‐HF^84^, ^85^	S/V vs. valsartan	HFpEF (*N* = 4796)	LVEF ≥45%	Prespecified kidney outcome (eGFR reduction ≥50%, ESKD, kidney‐related death): diabetes, HR, 0.42 (95% CI, 0.19–0.91); no diabetes, HR, 0.54 (95% CI, 0.33–0.89); *p‐*interaction = 0.59 Change in eGFR (mL/min/1.73 m^2^)/year: diabetes, S/V − 2.2 vs. valsartan −2.9; *P* = 0.001; no diabetes, S/V − 1.5 vs. valsartan −2.0; *P* = 0.006	Primary outcome *(*composite of CV death or hospitalization for HF): RR, 0.87 (95% CI, 0.75–1.01); *P* = 0.06 <60 eGFR (mL/min/1.73 m^2^) at baseline: HR, 0.79 (95%, CI 0.66–0.95); ≥60 eGFR (mL/min/1.73 m^2^) at baseline: HR, 1.01 (95% CI, 0.80–1.27), *P* = N/A Hospitalization for HF: RR, 0.85 (95% CI, 0.72–1.00)

ACEi, angiotensin‐converting enzyme inhibitor; ARB, angiotensin‐receptor blocker; aRR, adjusted risk ratio; CI, confidence interval; CKD, chronic kidney disease; CV, cardiovascular; CVD, cardiovascular disease; eGFR, estimated glomerular filtration rate; ESKD, end‐stage kidney disease; HF, heart failure; HFpEF, heart failure with preserved ejection fraction; HFrEF, heart failure with reduced ejection fraction; HR, hazard ratio; LVEF, left ventricular ejection fraction; N/A, not available; NS, not statistically significant; NT‐proBNP, N‐terminal pro‐brain natriuretic peptide; OR, odds ratio; RASi, renin–angiotensin system inhibitor; RR, risk ratio; S/V, sacubitril/valsartan; SE, standard error; T2D, type 2 diabetes; UACR, urine albumin‐to‐creatinine ratio.

^a^ Definitions of disease populations differ according to different trials.

^b^ For comparison of 150 mg vs. 50 mg losartan.

**Table 2:** Key findings of SGLT2i randomized clinical trials in patients with CKD or HF

Trial	Drug	Trial population (*N*)^a^	Key inclusion criteria	CKD outcomes (vs. placebo)	HF outcomes (vs. placebo)
*Participants with CKD*					
EMPA‐KIDNEY^60^	Empagliflozin	CKD (*N* = 6609)	eGFR ≥20 to <45 mL/min/1.73 m^2^ (irrespective of level of albuminuria) or eGFR ≥45 to <90 mL/min/1.73 m^2^ with UACR of ≥200 mg/g	Primary outcome (composite of kidney disease progression [ESKD, sustained eGFR of <10 mL/min/1.73 m^2^, sustained decline in eGFR ≥40%, kidney‐related death] or CV death): HR, 0.72 (95% CI, 0.64–0.82); *P* < 0.001 HF at baseline: HR, 1.00 (95% CI, 0.67–1.47), no HF at baseline: HR, 0.70 (95% CI, 0.61–0.80); *P* = N/A	Hospitalization for HF or CV death: HR, 0.84 (95% CI, 0.67–1.07); NS
DAPA‐CKD^61^, ^86^	Dapagliflozin	CKD (*N* = 4304)	eGFR 20 to 75 mL/min/1.73 m^2^ with a UACR of 200 to 5000 mg/g	Primary outcome (composite of sustained decline in eGFR ≥50%, ESKD, death from kidney or CV causes): HR, 0.61 (95% CI, 0.51–0.72); *P* < 0.001 HF at baseline: HR, 0.58 (95% CI, 0.37–0.91); no HF at baseline: HR, 0.62 (95% CI, 0.51–0.75); *P* = 0.59	CV death or hospitalization for HF: HR, 0.71 (95% CI, 0.55–0.92); *P* = 0.009 HF at baseline: HR, 0.68 (95% CI, 0.44–1.05); no HF at baseline: HR, 0.70 (95% CI, 0.51–0.97), *P* = 0.90
CREDENCE^62^, ^87^	Canagliflozin	Albuminuric CKD and T2D (*N* = 4401)	eGFR 30 to <90 mL/min/1.73 m^2^ with a UACR of >300 to 5000 mg/g	Primary outcome (composite of ESKD, a doubling of serum creatinine level for ≥30 days, or death from kidney or CV causes): HR, 0.70 (95% CI, 0.59–0.82); *P* = 0.00001 HF at baseline: HR, 0.89 (95% CI, 0.61–1.31); no HF at baseline: HR, 0.66 (95% CI, 0.55–0.79), *P* = 0.156	Hospitalization for HF: HR, 0.61 (95% CI, 0.47–0.80); *P* < 0.001 HF at baseline: HR, 0.76 (95% CI, 0.48–1.22); no HF at baseline: HR, 0.54 (95% CI, 0.39–0.75), *P* = 0.248
Nuffield/SMART‐C meta‐analysis^88^	SGLT2is	CKD (*N* = 25 898)	N/A	Kidney disease progression (a sustained ≥50% decrease in eGFR, a sustained low eGFR, ESKD, or death from kidney failure): RR, 0.62 (95% CI, 0.56–0.69)	CV death or hospitalization for HF: RR, 0.74 (95% CI, 0.66–0.82) for participants with diabetes (*n* = 20931); RR, 0.95 (95% CI, 0.65–1.40) for participants without diabetes (*n* = 4967)
SMART‐C meta‐analysis^89^	SGLT2is	CKD (*N* = 15 314)	N/A	–	MACE: HR, 0.87 (95% CI, 0.77–0.98) CV death: HR, 0.80 (95% CI, 0.68–0.95) HF death: HR, 0.41 (95% CI, 0.23–0.72)
*Participants with HF*					
EMPEROR‐Reduced^63^, ^90^	Empagliflozin	HFrEF (*N* = 3730)	LVEF ≤40%	Composite kidney outcome (ESKD or profound sustained reduction in eGFR): HR, 0.50 (95% CI, 0.32–0.77) Prevalent CKD at baseline: HR, 0.53 (95%, CI 0.31–0.91); no CKD at baseline: HR, 0.46 (95% CI, 0.22–0.99); *P* = 0.78	Primary outcome (composite of CV death or hospitalization for worsening HF): HR, 0.75 (95% CI, 0.65–0.86); *P* < 0.001 Prevalent CKD at baseline: HR, 0.78 (95% CI, 0.65–0.93); no CKD at baseline: HR, 0.72 (95% CI, 0.58–0.90); *P* = 0.63 Hospitalization for HF: HR, 0.70 (95% CI, 0.58–0.85); *P* < 0.001 Prevalent CKD at baseline: HR, 0.73 (95% CI, 0.57–0.94); no CKD at baseline: HR, 0.69 (95% CI, 0.51–0.93), *P* = 0.78
EMPEROR‐Preserved^64^, ^91^	Empagliflozin	HFpEF (*N* = 5988)	LVEF >40%	Composite kidney outcome (ESKD or profound sustained reduction in eGFR): HR, 0.95 (95% CI, 0.73–1.24) Prevalent CKD at baseline: HR, 0.97 (95% CI, 0.71–1.34); no CKD at baseline: HR, 0.92 (95% CI, 0.58–1.48); *P* = 0.8572 Change in eGFR (mL/min/1.73 m^2^)/year: −1.25 vs. −2.62; *P* < 0.001	Primary outcome (composite of CV death or hospitalization for worsening HF): HR, 0.79 (95% CI, 0.69–0.90); *P* < 0.001 Prevalent CKD at baseline: HR, 0.80 (95% CI, 0.69–0.94); no CKD at baseline: HR, 0.75 (95% CI, 0.60–0.95); *P* = 0.6682 Hospitalization for HF: HR, 0.73 (95% CI, 0.61–0.88); *P* < 0.001 Prevalent CKD at baseline: HR, 0.68 (95% CI, 0.54–0.86); no CKD at baseline: HR, 0.89 (95% CI, 0.66–1.21); *P* = 0.1667
DAPA‐HF^65^	Dapagliflozin	HFrEF (*N* = 4744)	LVEF ≤40%	Composite kidney outcome (ESKD, profound sustained reduction in eGFR, or death from kidney disease): HR, 0.71 (95% CI, 0.44–1.16)	Primary outcome (composite of CV death or worsening HF [hospitalization or urgent visit resulting in therapy]): HR, 0.74 (95% CI, 0.65–0.85); *P* < 0.001 <60 eGFR (mL/min/1.73 m^2^) at baseline: HR, 0.72 (95% CI, 0.59–0.86); ≥60 eGFR (mL/min/1.73 m^2^) at baseline: HR, 0.76 (95% CI, 0.63–0.92), *P* = NA Worsening HF: HR, 0.70 (95% CI, 0.59–0.83)
DELIVER^92^, ^93^	Dapagliflozin	HFmrEF/HFpEF (*N* = 6263)	LVEF >40%		Primary outcome (composite of CV death or worsening HF [hospitalization or urgent visit]): HR, 0.82 (95% CI, 0.73–0.92); *P* < 0.001 <45 eGFR (mL/min/1.73 m^2^) at baseline: HR, 0.93 (95% CI, 0.76–1.14), ≥45 to <60 eGFR (mL/min/1.73 m^2^) at baseline: HR, 0.68 (95% CI, 0.54–0.87); ≥60 eGFR (mL/min/1.73 m^2^) at baseline: HR, 0.84 (95% CI, 0.70–1.00); *P* = 0.16 Worsening HF: HR, 0.79 (95% CI, 0.69–0.91)
Nuffield/SMART‐C meta‐analysis^88^	SGLT2is	Stable HF (*N* = 20 721)	N/A		CV death or hospitalization for HF: RR, 0.77 (95% CI, 0.71–0.84) for participants with diabetes; RR, 0.78 (95% CI, 0.70–0.76) for participants without diabetes Results were consistent regardless of eGFR at baseline
SMART‐C meta‐analysis^89^	SGLT2is	HF (*N* = 20 725)	N/A		MACE: HR, 0.94 (95% CI, 0.86–1.01) CV death: HR, 0.88 (95% CI, 0.82–0.96) HF death: HR, 0.90 (95% CI, 0.75–1.07)

CI, confidence interval; CKD, chronic kidney disease; CV, cardiovascular; eGFR, estimated glomerular filtration rate; ESKD, end‐stage kidney disease; HF, heart failure; HFmrEF, heart failure with mildly reduced ejection fraction; HFpEF, heart failure with preserved ejection fraction; HFrEF, heart failure with reduced ejection fraction; HR, hazard ratio; LVEF, left ventricular ejection fraction; MACE, major adverse cardiovascular events; N/A, not applicable; NS, not statistically significant; RR, risk ratio; SGLT2i, sodium‐glucose cotransporter 2 inhibitor; T2D, type 2 diabetes; UACR, urine albumin‐to‐creatinine ratio.

^a^ Definitions of disease populations differ according to different trials.

**Table 3:** Key findings of MRA randomized clinical trials in patients with CKD or HF

Trial	Drug	Trial population (*N*)^a^	Key inclusion criteria	CKD outcomes (vs. placebo)	HF outcomes (vs. placebo)
*Participants with CKD*					
BARACK‐D^94^	Spironolactone	CKD (*N* = 1372)	eGFR 30–44 mL/min/1.73 m^2^	–	Composite of death, hospitalization for HF, stroke, HF, TIA or PAD, or first onset of any condition listed not present at baseline: HR, 1.05 (95% CI, 0.81–1.37)
FIGARO‐DKD^68^, ^95^	Finerenone	CKD (*N* = 7352)	eGFR ≥25 to <90 mL/min/1.73 m^2^ with a UACR of ≥30 to <300 mg/g or eGFR ≥60 mL/min/1.73 m^2^ with a UACR of ≥300 to ≤5000 mg/g	Composite kidney outcome (kidney failure, sustained decrease in eGFR ≥40% for 4 weeks, or death from kidney causes): HR, 0.87 (95% CI, 0.76–1.01)	Primary outcome (composite of CV death, nonfatal MI or stroke, or hospitalization for HF): HR, 0.87 (95% CI, 0.76–0.98); *P* < 0.03 With history of CVD: HR, 0.82 (95% CI, 0.70–0.96); without history of CVD: HR, 0.95 (95% CI, 0.77–1.17); *P* = N/A Hospitalization for HF: HR, 0.71 (95% CI, 0.56–0.90) With history of HF: HR, 0.70 (95% CI, 0.43–1.15); without history of HF: HR, 0.72 (95% CI, 0.55–0.95); *P* = 0.77
FIDELIO‐DKD^67^, ^96^	Finerenone	CKD (*N* = 5674)	eGFR ≥25 to <60 mL/min/1.73 m^2^ with a UACR of ≥30 to <300 mg/g OR eGFR ≥25 to <75 mL/min/1.73 m^2^ with a UACR of ≥300 to ≤5000 mg/g	Primary outcome (kidney failure, sustained decrease in eGFR ≥40%, or death from kidney causes): HR, 0.82 (95% CI, 0.73–0.93); *P* = 0.001 With history of CVD: HR, 0.70 (95% CI, 0.58–0.84); without history of CVD: HR, 0.94 (95% CI, 0.80–1.09); *P* = N/A	CV death, nonfatal MI, nonfatal stroke or hospitalization for HF: HR, 0.86 (95% CI, 0.75–0.99); *P* = 0.03 With history of CVD: HR, 0.85 (95% CI, 0.71–1.02); without history of CVD: HR, 0.86 (95% CI, 0.68–1.08); *P* = 0.85 Hospitalization for HF: HR, 0.86 (95% CI, 0.68–1.08)
FIDELITY^69^, ^97^	Finerenone	CKD (*N* = 13 026)	As per FIGARO‐DKD and FIDELIO‐DKD criteria	Composite of kidney failure, sustained decrease in eGFR ≥57% or death from kidney causes: HR, 0.77 (95% CI, 0.67–0.88); *P* = 0.0002 With history of CVD: HR, 0.71 (95% CI, 0.57–0.88); without history of CVD: HR, 0.81 (95% CI, 0.68–0.97); *P* = 0.3045	CV death, nonfatal MI, nonfatal stroke or hospitalization HF: HR, 0.86 (95% CI, 0.78–0.95); *P* = 0.0018 With history of CVD: HR, 0.83 (95% CI, 0.74–0.94); without history of CVD: HR, 0.91 (95% CI, 0.78–1.06), *P* = 0.35
*Participants with HF*					
RALES^98^	Spironolactone	Severe HFrEF (*N* = 1663)	LVEF ≤35%	–	Primary outcome (death from all causes): RR, 0.70 (95% CI, 0.60–0.82); *P* < 0.001 Hospitalization for HF: RR, 0.65 (95% CI, 0.54–0.77); *P* < 0.001
TOPCAT^70^	Spironolactone	HFpEF (*N* = 3445)	LVEF ≥45%	–	Primary outcome (death from CV causes, aborted cardiac arrest, or hospitalization for HF): HR, 0.89 (95% CI, 0.77–1.04); *P* = 0.14 <60 eGFR (mL/min/1.73 m^2^) at baseline: HR, 0.95 (95% CI, 0.77–1.17); ≥60 eGFR (mL/min/1.73 m^2^) at baseline: HR, 0.82 (95% CI, 0.66–1.02); *P* = 0.34 Hospitalization for HF: HR, 0.83 (95% CI, 0.69–0.99); *P* = 0.04
EPHESUS^99^	Eplerenone	Acute MI complicated by LV dysfunction and HF (*N* = 6632)	LVEF ≤40%	–	Primary outcome (CV death or hospitalization for CV events): RR, 0.87 (95% CI, 0.79–0.95); *P* = 0.002 Primary outcome (death from all causes): RR, 0.85 (95% CI, 0.75–0.96); *P* = 0.008
EMPHASIS‐HF^72^, ^100^	Eplerenone	HFrEF (*N* = 2737)	LVEF ≤30% (if >30 to 35%, a QRS duration of >130 ms on electrocardiography)	–	Primary outcome (CV death or hospitalization for HF): aHR, 0.63 (95% CI, 0.54–0.74); *P* < 0.001 <60 eGFR (mL/min/1.73 m^2^) at baseline: HR, 0.62 (95% CI, 0.49–0.79); *P =* 0.0001 CV death: aHR, 0.76 (95% CI, 0.61–0.94); *P* = 0.01 Hospitalization for HF: aHR, 0.58 (95% CI, 0.47–0.70); *P* < 0.001
FINEARTS‐HF^71^, ^101^	Finerenone	HFmrEF or HFpEF (*N* = 6001)	LVEF ≥40%	Composite kidney outcome (≥50% eGFR decline or kidney failure): HR, 1.33 (95% CI, 0.94–1.89) UACR: reduction, 30% (95% CI 25%–34%)	Primary outcome (composite of worsening HF events [unplanned hospitalization or urgent visit for HF] or CV death): rate ratio, 0.84 (95% CI, 0.74–0.95); *P* = 0.007 <60 eGFR (mL/min/1.73 m^2^) at baseline: HR, 0.91 (95% CI, 0.78–1.07); ≥60 eGFR (mL/min/1.73 m^2^) at baseline: HR, 0.72 (95% CI, 0.59–0.88), *P* = N/A Worsening HF events: rate ratio, 0.82 (95% CI, 0.71–0.94); *P* = 0.006 CV death: HR, 0.93 (95% CI, 0.78–1.11)
FINE‐HEART^73^	Finerenone	CKD (*N* = 18 991)	As per FINEARTS‐HF, FIDELIO‐DKD and FIGARO‐DKD	Composite kidney outcome (sustained decrease in eGFR ≥50% from baseline, sustained decline in eGFR to <15 mL/min/1.73 m^2^, kidney failure, or death due to kidney failure): HR, 0.80 (95% CI, 0.72–0.90); *P* < 0.001	CV death: HR, 0.89 (95% CI, 0.78–1.01); *P* = 0.076 History of CKD: HR, 0.84 (95%, CI 0.64–1.11); no history of CKD: HR, 0.90 (95% CI, 0.77–1.04); *P* = N/A Hospitalization for HF: HR, 0.83 (95% CI, 0.75–0.92); *P* < 0.001

aHR, adjusted hazard ratio; CI, confidence interval; CKD, chronic kidney disease; CV, cardiovascular; CVD, cardiovascular disease; eGFR, estimated glomerular filtration rate; HF, heart failure; HFmrEF, heart failure with mildly reduced ejection fraction; HFpEF, heart failure with preserved ejection fraction; HFrEF, heart failure with reduced ejection fraction; HR, hazard ratio; LV, left ventricular; LVEF, left ventricular ejection fraction; MI, myocardial infarction; MRA, mineralocorticoid receptor antagonist; N/A, not applicable; PAD, peripheral artery disease; RR, risk ratio; TIA, transient ischaemic attack; UACR, urine albumin‐to‐creatinine ratio.

^a^ Definitions of disease populations differ according to different trials.

**Table 4:** Key findings of GLP‐1 RA randomized clinical trials in patients with CKD or HF

Trial	Drug	Trial population (*N*)^a^	Key inclusion criteria	CKD outcomes (vs. placebo)	HF outcomes (vs. placebo)
*Participants with CKD*					
FLOW^53^	Semaglutide	CKD and T2D (*N* = 3533)	eGFR 25 to 75 mL/min/1.73 m^2^ with a UACR of >300 to <5000 mg/g if eGFR ≥50 mL/min/1.73 m^2^ or UACR of >100 to <5000 mg/g if eGFR 25 to <50 mL/min/1.73 m^2^	Primary outcome (composite of kidney failure, ≥50% reduction in eGFR, or death from kidney or CV causes): HR, 0.76 (95% CI, 0.66–0.88); *P* = 0.0003 History of chronic HF: HR, 0.67 (95% CI, 0.49–0.93), no history of chronic HF: HR, 0.79 (95% CI, 0.67–0.93); *P* = N/A	CV death: HR, 0.71 (95% CI, 0.56–0.89)
*Participants with HF*					
STEP‐HFpEF^75^	Semaglutide	HFpEF and obesity (*N* = 529)	LVEF ≥45%		Secondary outcome (hierarchical composite including death, HF events, and differences in the change in KCCQ‐CSS and 6MWD): win ratio, 1.72 (95% CI, 1.37–2.15); *P* < 0.001
STEP‐HFpEF DM^74^	Semaglutide	HFpEF, obesity, and T2D (*N* = 616)	LVEF ≥45%		Secondary outcome (hierarchical composite including death, HF events, and differences in the change in KCCQ‐CSS and 6MWD): win ratio, 1.58 (95% CI, 1.29–1.94); *P* < 0.001
Pooled STEP‐HFpEF and STEP‐HFpEF DM analysis^102^	Semaglutide	HFpEF, obesity, and T2D (*N* = 1145)	LVEF ≥45%		Secondary outcome (hierarchical composite including death, HF events, and differences in the change in KCCQ‐CSS and 6MWD): win ratio, 1.65 (95% CI, 1.42–1.91); *P* < 0.0001
SUMMIT^103^	Tirzepatide	HFpEF and obesity (*N* = 731)	LVEF ≥50%		Primary outcome (composite of CV death or worsening HF event [hospitalization for HF, intravenous therapy in an urgent care setting, or intensification of oral diuretic therapy]): HR, 0.62 (95% CI, 0.41–0.95); *P* = 0.026 Worsening HF events: HR, 0.54 (95% CI, 0.34–0.85)

6MWD, 6‐min walking distance; CI, confidence interval; CKD, chronic kidney disease; CV, cardiovascular; eGFR, estimated glomerular filtration rate; GLP‐1 RA, glucagon‐like peptide‐1 receptor agonist; HF, heart failure; HFpEF, heart failure with preserved ejection fraction; HR, hazard ratio; KCCQ‐CSS, Kansas City Cardiomyopathy Questionnaire clinical summary score; LVEF, left ventricular ejection fraction; N/A, not applicable; T2D, type 2 diabetes; UACR, urine albumin‐to‐creatinine ratio.

^a^ Definitions of disease populations differ according to different trials.

### RASis/ARNIs

#### Findings in participants with CKD

In RENAAL, which included participants with CKD who did not have HF at enrolment, losartan reduced the primary endpoint (doubling of the baseline serum creatinine concentration, end‐stage kidney disease [ESKD], or death; risk reduction, 16%; 95% CI, 2%–28%; *P* = 0.02) and reduced the first hospitalization for HF (risk reduction, 32%; *P* = 0.005) compared with placebo (*Table* *1*).^76^ Irbesartan reduced the rate of kidney‐related outcomes (primary endpoint: doubling of the baseline serum creatinine concentration, ESKD or death; adjusted risk reduction, 0.81; 95% CI, 0.67–0.99; *P* = 0.03) and the rate of hospitalization for HF (adjusted risk reduction, 0.77) compared with placebo in participants with nephropathy due to T2D in the IDNT study.^77^ A meta‐analysis of 119 trials including 64 768 participants with CKD reported that the use of angiotensin‐converting enzyme inhibitors or angiotensin‐receptor blockers, when compared with placebo, reduced kidney failure (odds ratio [OR], 0.61; 95% credible interval, 0.47–0.79 and OR, 0.70; 95% credible interval, 0.52–0.89, respectively) and reduced major CV events (OR, 0.82; 95% credible interval, 0.71–0.92 and OR, 0.76; 95% credible interval, 0.62–0.89, respectively) (*Table* *1*).^78^

HARP‐III (ISRCTN11958993), a randomized controlled trial that assessed ARNI therapy with sacubitril/valsartan versus irbesartan in participants with eGFR 20–60 mL/min/1.73 m^2^ did not show a significant difference in kidney endpoints (change in eGFR and UACR at 12 months).^79^ However, ARNI therapy did reduce NT‐proBNP compared with irbesartan (reduction, 18%; 95% CI, 11%–25%) (*Table* *1*).

#### Findings in participants with HF

In HEAAL (NCT00090259), high‐dose losartan (150 mg) significantly reduced the primary endpoint (death or hospitalization for HF; HR, 0.90; 95% CI, 0.82–0.99; *P =* 0.027) and hospitalization for HF (HR, 0.87; 95% CI, 0.76–0.98; *P* = 0.025) compared with low‐dose losartan (50 mg) in participants with HFrEF (*Table* *1*).^80^ In I‐PRESERVE (NCT00095238), which included participants with HFpEF, irbesartan did not significantly reduce the primary endpoint (death from any cause or hospitalization for CVD or the rate of hospitalization for HF) (*Table* *1*).^81^

In PARADIGM‐HF (NCT01035255), compared with enalapril, sacubitril/valsartan was associated with a significant reduction in the composite primary endpoint of CV death or hospitalization for HF (HR, 0.80; 95% CI, 0.73–0.87; *P* < 0.001) and hospitalization for HF (HR, 0.79; 95% CI, 0.71–0.89; *P* < 0.001) in participants with HFrEF, despite causing a modest increase in UACR. This finding was similar in participants with and without CKD at baseline.^83^ Sacubitril/valsartan, compared with valsartan, attenuated the decline of eGFR and reduced kidney outcomes in participants with HFpEF with diabetes (HR, 0.54; 95% CI, 0.33–0.89) and without diabetes (HR, 0.42; 95% CI, 0.19–0.91) in PARAGON‐HF (NCT01920711) (*Table* *1*).^84^, ^85^ Overall, the primary outcome (composite of CV death or hospitalization for HF) was not significantly reduced in the sacubitril/valsartan group compared with valsartan (*Table* *1*).^84^

### SGLT2is

#### Findings in participants with CKD

In EMPA‐KIDNEY (NCT03594110), in participants with CKD at risk of progression, empagliflozin decreased the primary outcome (composite of kidney disease progression [ESKD, a sustained eGFR of <10 mL/min/1.73 m^2^, sustained decline in eGFR ≥40%, death from kidney causes] or CV death); HR, 0.72; 95% CI, 0.64–0.82; *P* < 0.001). This effect was consistent regardless of the presence or absence of HF at baseline. Empagliflozin did not significantly reduce the composite of hospitalization for HF or CV death (*Table* *2*).^60^ Dapagliflozin reduced the composite of kidney disease progression, kidney death or CV death (HR, 0.61; 95% CI, 0.51–0.72; *P* < 0.001) and the composite of CV death or HF hospitalization (HR, 0.71; 95% CI, 0.55–092; *P* = 0.009) in participants with CKD in DAPA‐CKD (NCT03036150). The results were similar in patients with and without HF at baseline (*Table* *2*).^61^, ^86^ In CREDENCE (NCT02065791), canagliflozin reduced the primary composite kidney outcome (ESKD, doubling of the serum creatinine level, or kidney‐related or CV death; HR, 0.70; 95% CI, 0.59–0.82; *P* < 0.001) and hospitalization for HF (HR, 0.61; 95% CI, 0.47–0.80; *P* < 0.001) in participants with T2D and albuminuric CKD, regardless of HF at baseline.^62^, ^87^ The Nuffield Department of Population Health Renal Studies and SMART‐C meta‐analysis of SGLT2i studies demonstrated that SGLT2is reduced the risk of kidney disease progression in participants with CKD and diabetes (RR, 0.62; 95% CI, 0.56–0.69).^88^ The rate of CV death and HF hospitalization was reduced in participants treated with SGLT2is compared with placebo (RR, 0.74; 95% CI, 0.66–0.82; *Table* *2*). A subsequent SMART‐C meta‐analysis demonstrated that SGLT2is reduced CV death (HR, 0.80; 95% CI, 0.68–0.95) and HF‐related death (HR, 0.41; 95% CI, 0.23–0.72) in participants with CKD (*Table* *2*).^89^

#### Findings in participants with HF

In EMPEROR‐Reduced (NCT03057977), empagliflozin reduced the composite of CV death or HF hospitalization (HR, 0.75; 95% CI, 0.65–0.86; *P* < 0.001) and hospitalization for HF (HR, 0.70; 95% CI, 0.58–0.85; *P* < 0.001) in participants with HFrEF.^90^ The composite kidney outcome was halved in participants receiving empagliflozin compared with placebo (HR, 0.50; 95% CI, 0.32–0.77). These effects were consistent in patients with and without prevalent CKD at baseline.^90^ For participants with HFpEF in EMPEROR‐Preserved (NCT03057951), empagliflozin reduced the composite of CV death or HF hospitalization (HR, 0.79; 95% CI, 0.69–0.90; *P* < 0.001) as well as hospitalization for HF (HR, 0.73; 95% CI, 0.61–0.88; *P* < 0.001). Similar to EMPEROR‐reduced, these effects were independent of prevalent CKD status at baseline. The rate of eGFR decline was slower in the empagliflozin group compared with placebo, but the rate of the composite kidney outcome did not differ between the two treatment groups (*Table* *2*).^91^

Dapagliflozin reduced the rate of the composite of CV death or worsening HF (including hospitalization or need for urgent visit requiring intravenous therapy for HF) in DAPA‐HF (NCT03036124), regardless of eGFR at baseline (HR, 0.74; 95% CI, 0.65–0.85; *P* < 0.001). Dapagliflozin also reduced the rate of worsening HF (HR, 0.70; 95% CI, 0.59–0.83).^65^ DELIVER (NCT03619213) assessed the effect of dapagliflozin on clinical outcomes in participants with HFmrEF or HFpEF.^92^ The primary composite outcome (CV death or worsening HF) and worsening HF were both reduced (HR, 0.82; 95% CI, 0.73–0.92; *P* < 0.001 and HR, 0.79; 95% CI, 0.69–0.91, respectively) in the dapagliflozin group compared with the placebo group. There was no effect of eGFR at baseline on the primary outcome (*Table* *2*).^92^, ^93^

The Nuffield Department of Population Health Renal Studies and SMART‐C meta‐analysis demonstrated that use of a SGLT2i reduced the rate of CV death or HF hospitalization in participants with stable HF with diabetes (RR, 0.77; 95% CI, 0.71–0.84) and without diabetes (RR, 0.78; 95% CI, 0.70–0.76), regardless of eGFR status at baseline.^88^ The SMART‐C meta‐analysis demonstrated that SGLT2is reduced CV in participants with HF (HR, 0.88; 95% CI, 0.82–0.96; *Table* *2*).^89^

### MRAs

The steroidal MRAs spironolactone and eplerenone are approved for improving survival in patients with HFrEF.^104^, ^105^ Randomized clinical trials with steroidal MRAs have found little evidence of MRAs being effective for improving clinical outcomes in HFpEF^70^, ^106^, ^107^ or for slowing CKD progression.^108^, ^109^, ^110^ Spironolactone did not reduce CV outcomes in participants with stage IIIb CKD in the BARACK‐D trial (*Table* *3*) and was frequently discontinued due to safety concerns.^94^ However, it is important to note limitations of this study that may affect the interpretation of its findings, including that BARACK‐D enrolled participants with CKD with minimal albuminuria (median UACR, 1.5 mg/mmol [13.3 mg/g]), and that only 73% of participants contributed data at the 3‐year follow‐up mark.^94^

The nonsteroidal MRA finerenone demonstrates increased selectivity for the MR, a shorter half‐life, and different transcriptional coactivators compared with spironolactone and eplerenone (*Table* *3*).^40^, ^111^, ^112^

#### Findings in participants with CKD

Finerenone attenuates disease progression in patients with diabetic kidney disease and reduces CV events in this population, mainly driven by a reduction in HF hospitalizations.^95^, ^96^, ^97^ In FIGARO‐DKD (NCT02545049), the rate of the composite kidney outcome was reduced by finerenone compared with placebo (HR, 0.87; 95% CI, 0.76–1.01).^95^ Finerenone reduced the primary outcome (CV death, nonfatal myocardial infarction, nonfatal stroke or hospitalization for HF; HR, 0.87; 95% CI, 0.76–0.98; *P* < 0.03) and hospitalization for HF (HR, 0.71; 95% CI, 0.56–0.90) compared with placebo. The effect of finerenone on the primary endpoint and hospitalization for HF was consistent irrespective of a history of CVD and HF, respectively (*Table* *3*).^68^, ^95^ FIDELIO‐DKD (NCT02540993) assessed the effect of finerenone on a composite of kidney outcomes as a primary outcome and observed that finerenone reduced the primary outcome compared with placebo (HR, 0.82; 95% CI, 0.73–0.93; *P* = 0.001).^96^ The rates of CV death or hospitalization for HF were lower in the finerenone group than the placebo group (HR, 0.86; 95% CI, 0.75–0.99; *P =* 0.03). These results were similar in patients with and without CVD at baseline.^67^, ^96^ The prespecified FIDELITY analysis, which pooled data from FIGARO‐DKD and FIDELIO‐DKD, demonstrated a reduction in kidney outcomes (HR, 0.77; 95% CI, 0.67–0.88; *P =* 0.0002) and adverse CV events (HR, 0.86; 95% CI, 0.78–0.95; *P =* 0.0018).^97^ Of note, participants with HFrEF were excluded in these studies. However, subanalyses demonstrated a consistent effect of finerenone on these outcomes regardless of CVD at baseline (*Table* *3*).^69^, ^113^

In the phase 2 CONFIDENCE (NCT05254002) trial, initiation of finerenone and SGLT2 inhibition with empagliflozin in patients with CKD and T2D reduced UACR to a greater extent than either therapy alone.^135^ FINE‐ONE (NCT05901831) is an ongoing phase 3 study assessing the effect of finerenone on reducing UACR in participants with CKD and type 1 diabetes.^114^

MRAs have been associated with increased risk of hyperkalaemia due to the role of aldosterone in controlling potassium homeostasis.^115^ The effects of spironolactone and finerenone on the risk of hyperkalaemia were compared in a *post hoc* analysis of participants in the AMBER trial and in participants in FIDELITY that met the eligibility criteria for AMBER (CKD, T2D and treatment‐resistant hypertension).^116^ The incidence of hyperkalaemia at 12 weeks was 12% with finerenone (vs. 3% for placebo), 64% with spironolactone alone and 34% with spironolactone plus a potassium binder. The rate of treatment discontinuation due to hyperkalaemia was 0.3% with finerenone (vs. 0% for placebo), and 23% and 7% for spironolactone with or without a potassium binder, respectively.

#### Studies in participants with HF

Spironolactone reduced death from all causes (primary outcome; HR, 0.70; 95% CI, 0.60–0.82; *P <* 0.001) and hospitalization for HF (HR, 0.65; 95% CI, 0.54–0.77; *P <* 0.001) in participants with severe HFrEF in the RALES study (*Table* *3*). In contrast, in the TOPCAT (NCT00094302) trial involving participants with HFpEF, spironolactone demonstrated no significant reduction in the primary outcome (CV death, aborted cardiac arrest or hospitalization for HF), regardless of eGFR at baseline; however, hospitalization for HF was reduced with spironolactone compared with placebo (HR, 0.83; 95% CI, 0.69–0.99; *P* = 0.04; *Table* *3*).^70^

The EPHESUS study demonstrated that eplerenone reduced the rate of death from all causes (HR, 0.85; 95% CI, 0.75–0.96; *P* = 0.008) and the rate of CV death or hospitalization for HF (HR, 0.87; 95% CI, 0.79–0.95; *P* = 0.002) in participants with acute myocardial infarction complicated by left ventricular dysfunction and HF (*Table* *3*).^99^ Eplerenone reduced the primary composite of CV death or hospitalization for HF (adjusted HR, 0.63; 95% CI, 0.54–0.74; *P* < 0.001) as well as the components of the primary outcome compared with placebo in participants with HFrEF and mild symptoms of HF in EMPHASIS‐HF (NCT00232180) (*Table* *3*).^100^

HF outcomes trials with spironolactone and eplerenone indicate that they do not delay the progression of CKD.^108^, ^109^, ^110^ A pooled analysis of the RALES and EMPHASIS‐HF trials demonstrated that participants with a deterioration of eGFR to <30 mL/min/1.73 m^2^ after randomization had more than a two‐fold higher risk of the primary outcome (composite of CV death and hospitalization for HF) than participants without eGFR deterioration; however, the risk reduction with MRA therapy was consistent across participants with and without eGFR decline (*Table* *3*).^117^

In FINEARTS‐HF (NCT04435626), finerenone reduced the primary outcome (composite of total HF events [unplanned hospitalization or urgent visit for HF] or CV death) regardless of eGFR at baseline in participants with HFpEF or HFmrEF (HR, 0.84; 95% CI, 0.74–0.95; *P =* 0.007; *Table* *3*). Finerenone also reduced total HF events (first and recurrent) compared with placebo (HR, 0.82; 95% CI, 0.71–0.94; *P* = 0.006).^71^ There was no evidence that finerenone reduced the kidney composite endpoint (a sustained ≥50% decrease in eGFR, a sustained decline in eGFR <15 mL/min/1.73 m^2^, or long‐term dialysis or kidney transplantation) compared with placebo; however, UACR was reduced.^101^ To note, almost half the participants in this study had eGFR <60 mL/min/1.73 m^2^ at baseline.^71^ In the prespecified FINE‐HEART analysis, which pooled data from FIDELIO‐DKD, FIGARO‐DKD and FINEARTS‐HF, the primary endpoint of CV death occurred in 4.4% of participants in the finerenone group and in 5.0% of participants in the placebo group (HR, 0.89; 95% CI, 0.78–1.01; *P* = 0.076) and this finding was observed irrespective of CKD at baseline (*Table* *3*). Hospitalization for HF was reduced by finerenone (HR, 0.83; 95% CI, 0.75–0.92; *P* < 0.001; *Table* *3*).^73^

In addition to FINEARTS‐HF, several studies are also ongoing to further evaluate the efficacy of finerenone in participants with HF, including REDEFINE‐HF (NCT06008197), which includes participants with HFpEF hospitalized for acute decompensated HF; CONFIRMATION‐HF (NCT06024746), which will assess finerenone in combination with a SGLT2i in participants hospitalized for HF; and FINALITY‐HF (NCT06033950), which includes participants with HFrEF who are intolerant or not eligible for treatment with steroidal MRAs.^118^, ^119^, ^120^

In the TOPCAT and EPHESUS studies, MRAs were associated with a higher incidence of hyperkalaemia compared with placebo.^70^, ^99^ However, in EMPHASIS‐HF, hospitalization for hyperkalaemia did not differ between the eplerenone and placebo groups.^100^ There was also no significant difference in the incidence of serious hyperkalaemia (serum potassium ≥6.0 mmol/L) between treatment groups in the RALES study.^98^ A meta‐analysis of TOPCAT, EMPHASIS‐HF, RALES and the FINEARTS‐HF study concluded that the risk of hyperkalaemia with MRAs was doubled compared with placebo, but the absolute risk of serious hyperkalaemia was low.^121^ There was little heterogeneity in the risk of any hyperkalaemia across EF‐stratified trials.^121^

### GLP‐1 RAs

#### Studies in participants with CKD

Recently, the FLOW study demonstrated that semaglutide reduced the rate of the primary kidney outcome (composite of kidney failure, ≥50% reduction in eGFR, or death from kidney or CV causes; HR, 0.76; 95% CI, 0.66–0.88; *P =* 0.0003) and reduced CV death (HR, 0.71; 95% CI, 0.56–0.89) in participants with T2D and CKD. The effect of semaglutide on the primary kidney outcome was similar in patients with and without chronic HF at baseline (*Table* *4*).^53^ Although creatinine levels are affected by the loss of body mass that is associated with GLP‐1 RAs,^47^ the creatinine‐based and non‐creatinine‐based (cystatin C) eGFR assessments in the FLOW study were consistent.^53^ The authors noted that the kidney protective effects of semaglutide in FLOW were likely multifactorial and may include decreased inflammation, fibrosis and oxidative stress.^53^

#### Studies in participants with HF

The effect of semaglutide on a composite outcome including HF events was assessed as a secondary endpoint in the STEP‐HFpEF study in participants with HFpEF and obesity. An improvement in the composite outcome (win ratio, 1.72; 95% CI, 1.37–2.15; *P* < 0.001) was reported with semaglutide compared with placebo (*Table* *4*).^75^ Additionally, in the STEP‐HFpEF DM study of patients with obesity‐related HFpEF and T2D, semaglutide was associated with more wins in the composite endpoint (win ratio, 1.58; 95% CI, 1.29–1.94; *P* < 0.001), and greater reductions in HF symptoms and physical limitations, and greater weight loss compared with placebo at 1 year (*Table* *4*).^76^ The pooled analysis of both STEP‐HFpEF and STEP‐HFpEF DM also demonstrated a significant reduction in the composite endpoint (win ratio, 1.65; 95% CI, 1.42–1.91; *P* < 0.0001; *Table* *4*).^102^

In the SUMMIT trial, tirzepatide, a GLP‐1 RA and glucose‐dependent insulinotropic polypeptide receptor agonist, reduced the risk of the composite of CV death and worsening HF events (defined as hospitalization or need for urgent visit requiring intravenous therapy for HF, or intensification of oral diuretic therapy) compared with placebo in participants with HFpEF and obesity (HR, 0.62; 95% CI, 0.41–0.95; *P* = 0.026; *Table* *4*).^103^ The risk of worsening HF events was lower in the tirzepatide group compared with the placebo group (HR, 0.54; 95% CI, 0.34–0.85).^103^

## Challenges and opportunities in management of HF in patients with CKD

CKD is often underdiagnosed in HF, and HFpEF is underdiagnosed in CKD because of an overlap of symptoms, the challenges of accurate determination of volume status,^122^ and an underuse of screening tests in clinical practice.^25^, ^26^, ^27^, ^28^ Therefore, the development of a more personalized approach, using molecular profiling tools such as genetic markers, or biomarker profiles may prove useful in improving the accuracy of diagnosis and optimizing treatment plans.^123^ Regular monitoring of both cardiac and kidney parameters, and the use of imaging techniques, in patients at risk of comorbid CKD and HF also have the potential to improve early diagnosis.^124^

Treatment of patients with CKD and HF is complicated by the potential for adverse drug interactions between CKD and HF medications, reduced tolerance to RASis and beta‐blockers, diuretic resistance, and impaired efficacy of implantable cardioverter defibrillator and resynchronization device therapy.^125^, ^126^, ^127^ The introduction of a diuretic, whilst reducing the burden on the heart may increase the burden on the kidney and in some cases may reduce kidney edema. In these circumstances, careful consideration should be given to the diuretic used with loop diuretics seen as a potential option.^128^, ^129^ Treatment of CKD and HF may also be complicated by drug‐dosing challenges resulting from altered pharmacokinetics in these patients^125^ that may necessitate individualized medication titration strategies. In addition, clinical trial evidence of the use of therapies in the CKD with HF patient population is limited.^125^ Personalized treatment is an important future goal for improving outcomes and overcoming some of the current challenges of treating CKD and HF^123^; however, there are no current recommendations or algorithms to guide treatment decisions.

Patients with both CKD and HF are typically elderly and frail with high rates of mortality and hospitalization, and management is further complicated by the presence of additional comorbidities.^3^, ^130^, ^131^ Almost all patients with HF (98%) have at least one comorbidity, and 40% have CKD.^3^ A higher number of comorbidities is associated with an increased mortality rate as well as a requirement for a multidisciplinary approach to treatment.^3^

The presence of comorbidities constitutes a need for polypharmacy, which is associated with poor prognoses in people with HF.^132^ Increased risk of inappropriate prescription, poor adherence, and a higher risk of drug interactions are issues surrounding polypharmacy resulting in underuse of guideline‐recommended medications.^132^ For example, steroidal MRAs are underused in HFrEF despite clinical trial evidence of their efficacy, possibly related to concerns regarding risk of hyperkalaemia and worsening kidney function.^133^

A multidisciplinary approach to co‐ordination of care, including integrated cardio‐kidney clinics is a useful strategy to improve tailored treatment for patients with CKD and HF.^124^, ^126^ The use of standardized protocols for risk stratification, early intervention and use of guideline‐directed medical therapy are recommended^12^, ^30^ and their use may improve the dual management of CKD and HF, including newer agents such as SGLT2is and finerenone. Enhanced patient education programmes and remote monitoring systems may be utilized to improve medication adherence.^12^, ^30^

*Table* *5* highlights evidence gaps and opportunities for improving the management of patients with CKD and HF.

**Table 5:** Evidence gaps and future directions in the management of patients with CKD and HF

Unanswered questions	Future direction
**Diagnosis**	
How can early diagnosis of CKD be improved in patients with HF?	Develop a novel framework for kidney function evaluation and monitoring to more comprehensively evaluate kidney function at baseline and follow‐up in patients with HF
How can we predict development of HF in an individual person with CKD?	Develop predictive biomarkers, for example, based on proteomics
**Pharmacotherapy**	
How should newer therapies like nonsteroidal MRAs and GLP‐1 RAs be implemented clinically?	Conduct head‐to‐head trials and further research into pillared treatment approaches in patients with CKD and HF to inform patient care pathways; collect real‐world data in patients with CKD and HF
How can we predict the effect of drug initiation on kidney function in an individual person?	Collate data from clinical trials, observational studies, and real‐world clinical practice to develop predictive models
How should response to therapy be evaluated?	Investigate the value of a combination of various imaging techniques and biomarker assessments to monitor therapy response and disease progression
When should therapy for CKD be escalated in patients with HF?	Conduct longer‐term studies on the impact of therapy escalation at different disease stages
How can combination therapy be optimized?	Close monitoring of people receiving combination therapy to assess responses and develop approaches to optimization
How can we tailor therapies to individuals?	Explore kidney and neurohormonal responses to different therapies from multiple angles
How can we minimize polypharmacy?	Conduct head‐to‐head studies
Is pharmacotherapy efficacious in patients with HF and advanced CKD (stages IV–V)?	Perform studies in patients with late‐stage disease

CKD, chronic kidney disease; GLP‐1 RA, glucagon‐like peptide‐1 receptor agonist; HF, heart failure; MRA, mineralocorticoid‐receptor antagonist.

## Conclusions

CKD and chronic HF share common risk factors, pathophysiology and treatment targets.^33^, ^134^ Early assessment of kidney function in patients with HF is important to facilitate prompt intervention to slow CKD progression and reduce the risk of CV events, such as HF. Assessment for HF in patients with CKD is also necessary to optimize pharmacotherapy and clinical outcomes.^12^, ^30^ A number of guideline‐directed medical therapies are recommended to enhance clinical outcomes in patients with CKD and chronic HF.^12^, ^29^, ^30^, ^50^, ^51^

## Funding

This review was supported by Bayer AG. The authors wrote the paper independently with the assistance of a medical writer, who was funded by the sponsor. The sponsor is also the manufacturer of finerenone.

## Conflict of interest

JB has received honoraria for lectures/consulting with Amgen, AstraZeneca, Bayer, Boehringer Ingelheim, Bristol Myers Squibb, Cardior, Corvia, CVRx, Edwards, Norgine, Novartis, Pfizer, and Roche Vifor; and research support for their department from Abiomed, CVRx, Norgine, Roche, and Zoll. ETK reports receiving research funding/consulting fees/lecture fees from Astellas, AstraZeneca, Bayer, Boehringer Ingelheim, Bristol Myers Squibb, Daiichi‐Sankyo, Eli Lilly Japan KK, Menarini, MSD KK, Novo Nordisk, Ono Pharmaceutical, Pfizer, Takeda, and Tanabe‐Mitsubishi; and research funding from Abbott, Ono Pharmaceutical, and Tanabe‐Mitsubishi. JR reports personal fees from Amgen, AstraZeneca, Bayer, Boehringer Ingelheim, Bristol Myers Squibb, Daiichi‐Sankyo, MSD KK, Pfizer, Takeda, and Tanabe‐Mitsubishi; grants from Abbott Japan; and grants and personal fees from Ono Pharmaceutical.
